# Neoadjuvant Treatment in Resectable Pancreatic Cancer. Is It Time for Pushing on It?

**DOI:** 10.3389/fonc.2022.914203

**Published:** 2022-05-30

**Authors:** Marco Vivarelli, Federico Mocchegiani, Daniele Nicolini, Andrea Vecchi, Grazia Conte, Enrico Dalla Bona, Roberta Rossi, Andrea Benedetti Cacciaguerra

**Affiliations:** Hepato-Pancreato-Biliary and Transplant Surgery, Department of Experimental and Clinical Medicine, Polytechnic University of Marche, Ancona, Italy

**Keywords:** pancreatic adenocarcinoma, resectable pancreatic adenocarcinoma, neoadjuvant treatment, chemotherapy, upfront surgery

## Abstract

Pancreatic resection still represents the only curative option for patients affected by pancreatic ductal adenocarcinoma (PDAC). However, the association with modern chemotherapy regimens is a key factor in improving the inauspicious oncological outcome. The benefit of neoadjuvant treatment (NAT) for borderline resectable/locally advanced PDAC has been demonstrated; this evidence raises the question of whether even resectable PDAC should undergo NAT rather than upfront surgery. NAT may avoid futile surgery because of undetected distant metastases or aggressive tumor biology, providing more effective systemic control of the disease, which is hampered when adjuvant chemotherapy is delayed or precluded. However, recent data show controversial results regarding the efficacy and safety of NAT in resectable PDAC compared to upfront surgery. Although several prospective studies and meta-analyses indicate better oncologic outcomes after NAT, there are some biases, such as the methodological approaches used to capture the events of interest, which could make these results hardly reproducible. For instance, per-protocol studies, considering only the postoperative outcomes, tend to overestimate the performance of NAT by excluding patients who will never be suitable for surgery due to the development of chemotoxicity or tumor progression. To draw reliable conclusions, the studies should capture the events of interest of both strategies (NAT/upfront surgery) from the time of allocation to a specific treatment in an intention-to-treat fashion. This critical review highlights the current literature data concerning the use of NAT in resectable PDAC, summarizing the results of high-quality studies and focusing on the methodological issues of the most recent pieces of evidence.

## Introduction

Pancreatic ductal adenocarcinoma (PDAC) is the fourth and fifth most common cause of cancer deaths in the USA and Europe, respectively (1, 2). The incidence of pancreatic ductal adenocarcinoma has risen rapidly. By 2030, PDAC is expected to become the second most prevalent cause of death by cancer after lung cancer (3). Although surgery represents the only potentially curative treatment for PDAC, only 20% of patients are candidates for surgery because of the presence of distant metastasis or major vessel involvement at the time of the diagnosis (4). Based on the well-known radiological classification of PDAC, the National Comprehensive Cancer Network (NCCN) defines as resectable PDAC (RPDAC) tumors that do not show any contact with arteries (celiac axis, superior mesenteric artery, or common hepatic artery) or veins (the superior mesenteric vein or portal vein). If venous contact is present, this must involve ≤180° of the vessel circumference without any vein contour irregularity to qualify the tumor as resectable. Based on this radiological classification, the classification of PDAC has been standardized worldwide (5).

Neoadjuvant treatment (NAT) in PDAC is currently recommended by the International Guidelines for patients with borderline resectable or locally advanced disease, considering these neoadjuvant protocols as an induction therapy (6). In this subgroup of patients with advanced stage disease due to vascular involvement at the time of the diagnosis, the delivery of NAT takes over the task of testing the biological behavior of the tumor, decreasing the incidence of explorative surgery and downstaging disease in patients to achieve surgical resectability (7, 8). Thanks to the development of new effective chemotherapeutic protocols, namely gemcitabine and nab-paclitaxel (Abraxane) or leucovorin, 5-fluorouracil, irinotecan, oxaliplatin (FOLFIRINOX), postoperative oncologic outcomes of borderline resectable and even locally advanced PDAC have steadily improved and they are now comparable to those of patients with RPDAC at the time of the first diagnosis (9–12).

## Neadjiuvant Treatments in Resectable PDAC: Lights and Shadows

**Figure 1:**
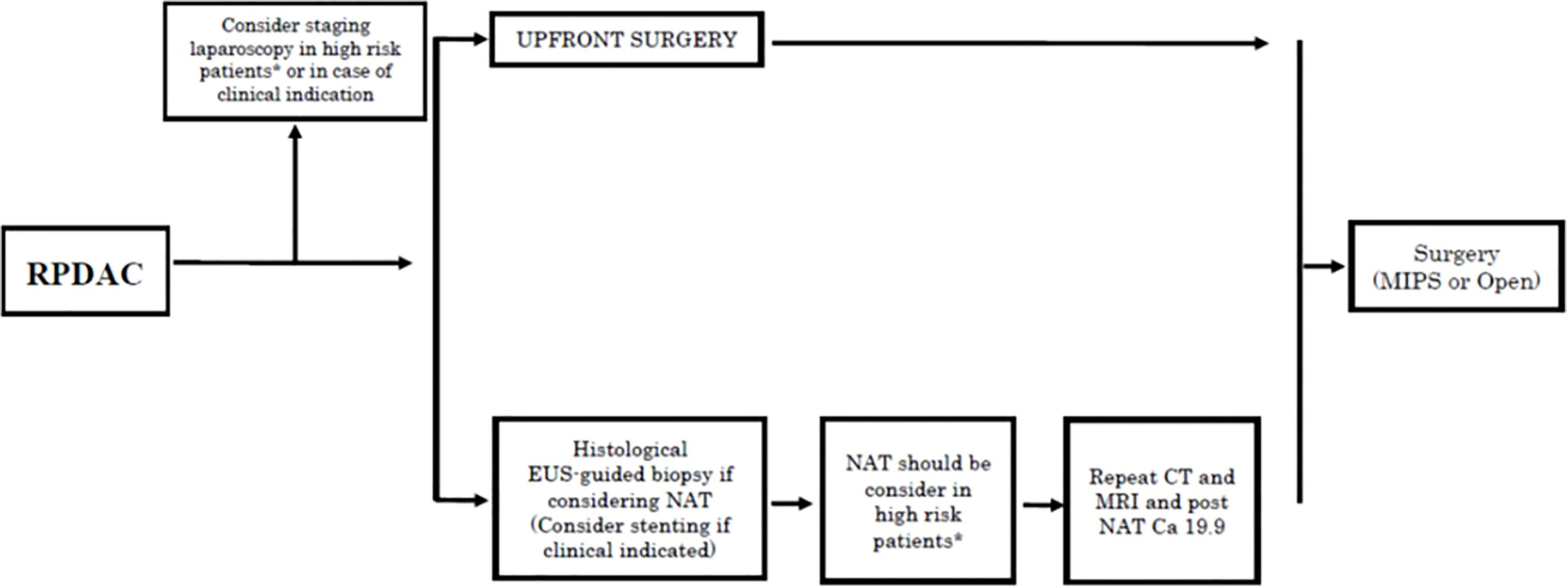
Treatment algorithms for resectable pancreatic cancer reported in the NCCN Guidelines Version 1.2021. (6). RPDC, Resectable pancreatic ductal adenocarcinoma; NAT, neoadjuvant treatments; EUS, endoscopic ultrasound; CT, computed tomography; MRI, Magnetic resonance; MIPS, minimally invasive pancreatic surgery. *High risk patients: Patiens with high risk features in terms of radiological (large primary tumor, large lymphonodes suspected for metastatic) and/or biological findings (Ca 19.9 > 500 U/ml in case of absence of biliary obstruction and/or cholangitis, extreme irradiated pain, excessive weight loss).

In the last two decades, up-front surgery (UFS) has not substantially changed the overall (OS) and disease-free survival (DFS) of patients with RPDAC, despite the consistent development of adjuvant therapy (AT). The presence of undetected micrometastases at the time of surgery together with the biological aggressiveness of the tumor itself are the main reasons for slipping into early tumor recurrence (13, 14). Based on clinical evidence, many experts have suggested that PDAC, even in its early-stage, should be considered as a systemic disease that could potentially benefit from NAT (15–19). The recent NCCN Guidelines Version 1.2021 recommended NAT not only in cases of borderline resectable pancreatic cancer but also in high-risk resectable PDAC (based on radiological findings, elevated CA 19-9, large tumors, large regional lymph nodes, excessive weight loss, and extreme pain) (**Figure 1**). However, evidence on the benefits of NAT in RPDAC is still weak, so in daily clinical practice, upfront-surgery followed by adjuvant chemotherapy is still recommended as the standard treatment in cases of PDAC judged as “resectable” (4, 6). Although this management is currently performed in clinical practice, many concerns still remain as a large proportion of resected patients develop early recurrence, nullifying the potential advantages of the UFS (20). Besides, pancreatic resection is still a high-risk procedure, and nearly 50% of resected patients eventually fail to receive adjuvant therapy due to post-operative complications or reduced performance status. These possible downsides of surgery strengthen the concept that NAT might be given to patients with RPDAC to detect aggressive disease by preventing futile surgical procedures, treat the potential hidden micrometastases, achieve a higher R0 resection rate, and deliver systemic therapy in all cases (21). Once the diagnosis of RPDAC is established, the choice of surgery as first-line treatment is no longer so obvious, as NAT might be considered as well.

Although studies have shown that NAT tends to improve the OS of patients with resectable PDAC, most of them were limited by the low level of evidence (retrospective cohort studies/case series), the small sample size, and older chemotherapy regimens used (22–24). Considering that several randomized controlled trials (RCT) failed to demonstrate a clear advantage in OS or did not provide the results in the specific subgroup of resectable patients, it has been necessary for researchers to rely on systematic reviews that pool the existing evidence (18, 25). Several meta-analyses recently reported favorable results regarding NAT in RPDAC, in terms of long-term survival and R0 resection rate. However, most of these studies were either flawed by substantial heterogeneity in terms of definition of resectability, chemo-radiotherapic regimens administered, or did not distinguish the results of resectable from borderline resectable PDAC. Moreover, we should consider that, when compared to those who underwent upfront-surgery, 30% of the patients who received NAT dropped-out from a surgical treatment program, and therefore did not receive any curative therapy (26–29).

Indeed, NAT in RPDAC patients may be related to potential drawbacks, such as the onset of jaundice, disease progression, and chemotherapy-related toxicity, leading to drop-out of the patient from the surgical plan (30). Theoretically, studies based on intention-to-treat (ITT) analysis may address these issues. In fact, ITT analysis considers the events of interest from the time of diagnosis and not from the time of surgical treatment.

A critical review should be performed even of those studies designed with an ITT perspective, to avoid misleading results produced by substantial methodological bias. For instance, in the recent meta-analysis reported by Versteijne et al. that showed a significant improvement in ITT-OS for RPDAC treated by NAT, several single-arm studies were included, which represents a significant reporting bias (31). In another recent meta-analysis by Van Dam et al., although the strict selection criteria (only RCT included) and the ITT methodology were used, the results focused mostly on borderline resectable tumors (32). As the role and true effectiveness of NAT in RPDAC remain unclear, in this critical review, we aimed to assess the benefits of NAT in patients with RPDAC compared with the standard practice, represented by upfront surgery. To minimize the selection bias, we decided to set the following inclusion criteria:

• Highest level of evidence studies:
 ○ RCT
 ○ The most recent metanalyses (2019–2021).
• Clear report of results of NAT in RPDAC (excludes those pooling together results of RPDAC and borderline resectable PDAC)
• ITT-based analysis
• Clear comparison between NAT and UFS for RPDAC.

## Key Studies Investigating NAT Effectiveness in RPDAC

Among papers analyzing the benefits of NAT in patients affected by RPDAC, eight reports matched the criteria to be considered valuable for this critical review (18, 24, 27, 33–37) (**Table 1**). These studies indicate contentious results on the advantages of NAT for RPDAC, especially in terms of OS and DFS. Conversely, wider agreement was found when looking at the resection rate and pathologic parameters (i.e., R0 rate and lymph node metastasis rate).

**Table 1:** Summary of the key studies assessing NAT effectiveness in RPDAC.

Authors	Year	Country	Study design	No. of patients	OS		DFS	RR	Pathological parameters	
					PP	ITT			R0	LN0
**Golcher et al. (** 18 **)**	2015	Germany	RCT*	73*	=	=	=	=	=	=
**Casadei et al. (** 37 **)**	2015	Italy	RCT*	38*	NR	=	NR	<NAT	=	=
**Reni et al. (** 24 **)**	2018	Italy	RCT**	93	>NAT	>NAT	NR	=	>NAT	>NAT
**Unno et al. (** 34 **)**	2019	Japan	RCT°	360	>NAT	>NAT	NR	=	>NAT	>NAT
**Lee et al. (** 35 **)**	2019	Korea	Meta-analysis	9691	>NAT	=	=	<NAT	>NAT	>NAT
**Pan et al. (** 27 **)**	2020	China	Meta-analysis	2286	>NAT	=	>NAT	<NAT	>NAT	>NAT
**Ye et al. (** 36 **)**	2020	China	Meta-analysis	9773	NR	=	=	<NAT	>NAT	>NAT
**Versteijine et al. (** 33 **)**	2020	Netherlands	RCT	246	NR	=	>NAT	=	=	>NAT

NAT, neoadjuvant treatments; OS, overall survival; DFS, disease-free survival; PP, per-protocol analysis; ITT, intention-to-treat analysis; RR, resection rate; R0, negative margin; LN0, negative metastatic lymph nodes; >NAT, advantage in NAT patients; NR, not reported; =, comparable results between NAT and UFS.

*Concluded earlier due to the slow recruitment.

**Due to the modifications in the standard of care for adjuvant therapy regimens, phase 3 of the PACT-15 was suspended.

°Preliminary results presented at the 2019 ASCO Gastrointestinal Cancers Symposium.

### Patients Survival

In a recent meta-analysis reported by Pan et al., 17 studies investigating the effectiveness of NAT for PDAC from 2011 to 2018 were included; however, only 9 of them focused specifically on RPDAC, while the others combined results obtained from studies on both RPDAC and border-line resectable PDAC (27). The per-protocol (PP) analysis (outcome observed after curative surgery) showed better OS for patients who underwent NAT (HR, 0.75 [95% CI, 0.63–0.89], I2 = 0%), but this finding was not confirmed in the ITT-pooled analysis, which showed comparable results between the NAT and the UPS group (HR, 1.02 [95% CI, 0.85–1.22], I2 = 26.5%). Of note, although in this study RPDAC patients undergoing NAT presented a trend toward better DFS and a lower recurrence rate than those of the UFS group, this finding failed to achieve statistical significance (DFS: HR = 0.80, P = 0.137; recurrence rate: OR = 0.77, P = 0.131). Among the studies analyzed in this systematic review, Golcher et al. published in 2015 the first RCT on NAT for RPDAC, reporting comparable results to UFS in terms of OS and DFS (18). The study was stopped earlier than planned due to the slow recruitment (only 73 patients were recruited between 2003 and 2009) and the chemotherapy regimens used look outdated nowadays, making these results unreliable. Similarly, Casadei et al. in their RCT published in 2015 reported comparable OS between NAT and UFS; however, as with the aforementioned trial, this study was concluded earlier due to the difficulty in recruiting patients (only 38 were eventually recruited) (37) and the old chemotherapy regimens used represent a possible limitation again, as gemcitabine alone is actually outdated in favor of FOLFIRINOX or gemcitabine and Abraxane regimens.

In the same way, Lee et al. in their systematic review compared the OS of the two treatment strategies (NAT *vs* UFS) in RPDAC patients by stratifying the results according to the analytic methods (ITT *or* PP) (35). Interestingly, the authors performed a sensitivity analysis to investigate the sources of heterogeneity, making this report one of the most reliable from a methodological perspective. In the studies reviewed until 2018, as already reported by Pan et al., 12 PP analysis papers showed that NAT brought a survival benefit over UFS (HR 0.72, 95% CI 0.68–0.76, P <0.001), whereas the 7 studies conducted with ITT methodology did not show any statistical difference (HR 0.96, 95% CI 0.82–1.12, P = 0.610). When considering only patients in whom the anticancer therapy was effectively delivered (before or after surgery), PP-OS appeared significantly improved in the NAT strategy (HR 0.82, 95% CI 0.71–0.93, P = 0.003). However, from an ITT perspective, 36.3% of the patients in the NAT treatment strategy eventually failed to undergo surgery versus 17.3% of those who were deemed to have UFS, probably due to a significant increase in the so-called pre-surgical “attrition rate” in the NAT group. Attrition in surgery is defined as loss to follow-up secondary to self-discharge, inability to complete the therapeutic plan due to poor compliance or deterioration of the physical condition. When considering only patients who completed both surgery and chemotherapy, NAT showed a PP-OS advantage over UFS.

The PREOPANC, a Dutch randomized phase III trial of 16 centers, enrolling 246 patients with resectable or borderline resectable pancreatic cancer, was the first RCT to utilize preoperative chemoradiotherapy (33). In this study, the results obtained were substantially in keeping with the other studies previously described, with comparable OS in the ITT analysis. However, the application of the protocol used in this trial, namely, the use of single‐agent gemcitabine adjuvant therapy, appears somehow outdated currently. Moreover, the median OS in the UFS group was better than expected (14 instead of 11 months), which might be related to a substantial drop-out of high-risk patients (“presurgical triage”), as reported by the authors. The PREOPANC trial, as well as previous studies, when considering resectable patients only, did not demonstrate a significant change in OS and DFS of RPDAC patients; in contrast, a trend toward better survival was observed for the UPS arm. However, the advantages found in the R0 rate and positive lymph node rates might support NAT in RPDAC.

To date, only 2 studies have reported an advantage of NAT compared to UFS in terms of OS. Reni et al. (PACT-15) published in 2018 the results of a randomized, open-label, phase 2–3 trial: the trial had strict selection criteria and it was structured into three arms: two arms included patients undergoing UFS with two different adjuvant treatments, while the third arm included patients who received NAT (24). Median OS was 38.2 months (27.3–49.1) for patients randomly assigned to the NAT arm, and 20.4 (95% CI 14.6–25.8) and 26.4 months (95% CI 15.8–26.7) for patients randomly assigned to the 2 UFS groups. However, as mentioned by the authors, during phase 2 of the trial, the standard-of-care for adjuvant therapy changed and new chemotherapy regimens, which are apparently more active or based on more robust evidence than the PEXG regimen (second arm), were available only for the metastatic disease setting. Therefore, the authors decided to not proceed with phase 3 of the trial. Moreover, the sample size of each study arm was about 1/3 of the required population needed to statistically demonstrate the OS advantage of NAT over UFS.

Lastly, the Prep-02/JSAP05 is a Japanese randomized multi-institutional phase II/III trial that compared NAT using gemcitabine and S-1 (NAC-GS) with upfront surgery for patients with RPDAC (34). As a matter of fact, this study is the first multiinstitutional Phase III trial showing that NAT leads to significant advantages in terms of OS in patients with RPDAC in ITT analysis, with the preliminary results being presented at the 2019 ASCO Gastrointestinal Cancers Symposium. Unlike the previous papers reviewed, this study reported a median OS of 36.7 months in the NAT group and 26.6 months in the UFS group (p = 0.015; HR: 0.72; 95% CI: 0.55–0.94); patients in the NAT arm were treated with different therapeutic protocols with a longer duration of systemic therapy than those in the UFS arm, and these preliminary results have not been confirmed in a thorough report yet. Unfortunately, no significant conclusions can be drawn from the aforementioned preliminary results yet. Indeed, after more than three years since this report, no study has been published, raising some doubts about the completion of the trial itself.

### Resection Rate and Pathologic Parameters

Among the secondary outcomes, the two meta-analyses reviewed showed concordant results in terms of resection rate that was significantly lower in RPDAC patients undergoing NAT (27, 35). Noticeably, this finding was confirmed in the systematic review of Ye et al. that was mostly focused on these parameters: a significantly lower resection rate was observed in the NAT compared with the UFS group (OR = 2.18, 95% CI 1.41–3.37, P = 0.0004, I2 = 43%) regardless of the treatment protocols used. The authors concluded that NAT in patients with RPDAC may jeopardize the opportunity for surgical resection (36).

In the PREOPANC trial, the resection rate was 62% in the NAT arm and 72% in the UFS arm; however, this finding failed to reach statistical significance (P = .058). The Prep-02/JSAP05 and the PACT-15 trial did not show any difference in the resection rate, but the need for stronger evidence on this issue was recommended (24, 33, 34). A lower resection rate may not necessarily represent a downside of NAT; for some authors, NAT could in fact triage patients who would not benefit from surgery.

Concerning pathologic parameters, there is some evidence in all studies that a higher R0 resection rate and a lower rate of metastatic lymph nodes were recorded in NAT compared to UFS. For instance, recently, in the meta-analysis reported by Xu et al., patients who underwent NAT presented an increased R0 resection rate for RPDAC (OR = 1.59, 95% CI = 1.41–1.80) (38). However, when analyzing from an ITT perspective, this result failed to reach significance (OR = 1.45, 95% CI = 0.91–2.30). Notably, we decided to exclude this study from our review because the ITT methodology was assessed for one parameter only (R0 rate), thus failing to meet the inclusion criteria set in this review.

## Present Evidences and Future Perspectives

In this critical review, we aimed to reduce potential methodological biases of the available studies by evaluating the highest quality papers and the most recent systematic reviews reporting data on the use of NAT in RPDAC. Furthermore, we considered only studies based on ITT analysis instead of PP methodology because we strongly believe that ITT is the only analytic method able to capture and analyze all the events of interest (i.e., radio-chemotoxicity, unsuitability for surgery after NAT) from the diagnosis, thus demonstrating the real harms and benefits of new oncological approaches.

Nowadays, whereas there is robust evidence to support the systematic use of NAT in borderline resectable tumors, we are far from achieving a definitive agreement on the opportunity to offer NAT as the first-line treatment to all patients with RPDAC. The RCT published so far, comparing the two above-mentioned strategies, failed to demonstrate with statistical significance the advantage of NAT in terms of OS and DFS in patients with RPDAC (18, 24, 33, 37). The results of another Japanese RCT that seems to show improved survival in patients who underwent NAT for RPDAC have not been published in full yet, thus raising some doubts about the good completion of the trial (34).

In favor of NAT for RPDAC, there could be the feeling that the drop-out from surgery, which is higher when NAT is performed, should not be considered a missed chance of cure but an opportunity for sparing futile high-risk surgery. However, this assumption needs clear conformation based on evidence. Alternatively, a proportion of resectable patients could miss the chance of radical surgery due to the pre-surgical “attrition” and the disease progression during NAT. Furthermore, for patients with high bilirubin levels at the time of the diagnosis, there might be a considerable delay in starting the chemotherapy, as not all biliary stenting procedures achieve an immediate effect.

We believe that the definition of resectability based on technical features only (absence of tumor vascular involvement) does not capture those patients for whom NAT can have a strong rationale and that studies should pobably be more focused on high-risk resectable cancers with high levels of serum CA 19-9 or evidence of lymph node involvement.

In the future, the choice of the best multimodal treatment of RPDAC should probably be based on the biological behavior of the tumor rather than on the loco-regional staging of the tumor, which currently represents the cornerstone of the decision-making process with regard to first-line treatment. More effective and individualized systemic therapeutic regimens will probably stem from a better knowledge of clinic-pathological prognostic factors such as molecular profiling and novel biomarkers (39).

## Author Contributions

MV and ABC conceived the paper. MV, ABC, and RR wrote the manuscript. FM, AV, GC, EDB, and DN contributed critical revision of the manuscript for important intellectual content. All authors listed have made a substantial, direct, and intellectual contribution to the work and approved it for publication.

## Conflict of Interest

The authors declare that the research was conducted in the absence of any commercial or financial relationships that could be construed as a potential conflict of interest.

## Publisher’s Note

All claims expressed in this article are solely those of the authors and do not necessarily represent those of their affiliated organizations, or those of the publisher, the editors and the reviewers. Any product that may be evaluated in this article, or claim that may be made by its manufacturer, is not guaranteed or endorsed by the publisher.

## Abbreviations

NAT, Neoadjuvant treatment; UFS, Up-front surgery; RCT, randomized controlled trials; OS, Overall survival; DFS, Disease-free survival; PDAC, Pancreatic ductal adenocarcinoma; RPDAC, Resectable pancreatic ductal adenocarcinoma; NCCN, National Comprehensive Cancer Network; ITT, intention-to-treat; PP, per-protocol.
