# Supplementary figures and images for: Neoadjuvant Treatment in Resectable Pancreatic Cancer. Is It Time for Pushing on It?
Source: Front Oncol. 2022 May 30;12:914203. doi: 10.3389/fonc.2022.914203 (PMC9195424; doi:10.3389/fonc.2022.914203)

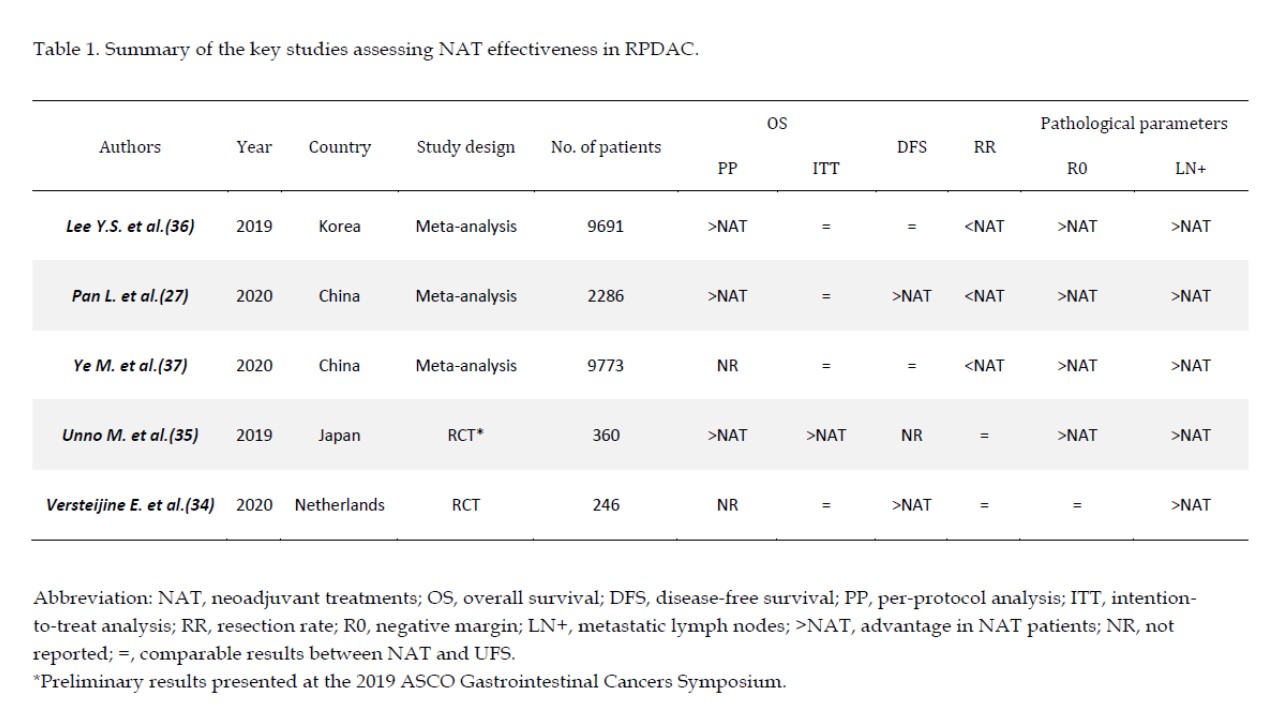

Supplement: Supplementary file 1 [file Image_1.jpeg]
